# Facilitators and barriers affecting the implementation of e-health for chronic respiratory diseases in remote settings: a qualitative evidence synthesis

**DOI:** 10.1186/s12913-024-12050-4

**Published:** 2025-01-04

**Authors:** Emil Matias Salmi, Francesca Wanda Basile, Faiz Ahmad Khan, Larry Watt, Rinn Song, Else Margreet Bijker

**Affiliations:** 1 Department of Paediatrics, Maastricht University Medical Center, MosaKids Children’s Hospital, Maastricht, the Netherlands; 2 Department of Paediatrics, Oxford Vaccine Group, University of Oxford, Oxford, UK; 3 Respiratory Epidemiology & Clinical Research Unit, Centre for Outcomes Research & Evaluation, Research Institute of the McGill University Health Centre and Respiratory Division, McGill University, Montreal, QC Canada; 4 Ungava Tulattavik Health Centre, Québec, Kuujjuaq Canada

**Keywords:** E-health, Telemonitoring, Respiratory disease, COPD, Asthma, Chronic, Implementation, MHealth

## Abstract

**Background:**

Chronic respiratory diseases are important causes of disability and mortality globally. Their incidence may be higher in remote locations where healthcare is limited and risk factors, such as smoking and indoor air pollution, are more prevalent. E-health could overcome some healthcare access obstacles in remote locations, but its utilisation has been limited. An improved understanding of barriers and facilitators to the implementation of e-health in remote locations could aid enhanced application of these approaches.

**Methods:**

We performed a qualitative evidence synthesis to explore factors affecting the successful implementation of e-health interventions in remote locations for patients with chronic respiratory diseases. We searched PubMed, CINAHL, Embase, Web of Science and PsycINFO databases for qualitative and mixed-methods studies. Studies were assessed by two researchers, and 41 studies were included in the synthesis. Quality was assessed via the CASP-tool. Findings were coded with Atlas.ti software and categorised based on an adapted Digital Health Equity Framework.

**Results:**

Nineteen themes were identified across five levels (individual, interpersonal, community, society and technology), with associated facilitators and barriers for implementation. An important facilitator of e-health was its role as a tool to overcome obstacles of distance and to increase access to care and patients’ self-efficacy. Potential barriers included the reduction of in-person interactions and an increased burden of work for healthcare providers. Good quality, usability, adaptability and efficacy of e-health interventions were important for implementation to be successful, as were adaptation to the local setting — including culture and language —and involvement of relevant stakeholders throughout the process.

**Conclusions:**

Several factors affecting the implementation of e-health in remote and rural locations for patients with chronic respiratory disease were identified. Intervention objectives, target population, geographical location, local culture, and available resources should be carefully considered when designing an e-health intervention. These findings can be used to inform the successful design and implementation of future e-health interventions.

**Supplementary Information:**

The online version contains supplementary material available at 10.1186/s12913-024-12050-4.

## Background

Chronic respiratory diseases are a leading cause of disability and mortality globally [1]. They include a range of infectious and non-infectious health conditions such as tuberculosis, chronic obstructive pulmonary disease (COPD), asthma, occupational lung diseases and pulmonary hypertension. As with many other chronic conditions, their prevalence is increasing [2, 3]. Despite being a heterogeneous group, chronic respiratory diseases share similar risk factors, such as smoking, air pollution, dust and occupational chemicals [4]. Early diagnosis, close monitoring, appropriate therapy, and easy access to health care can improve outcomes [5, 6].

Rurality may be a determinant of risk and outcome of chronic respiratory disease. For example, COPD incidence is higher in populations living in rural and remote areas, who are, on average, also more exposed to risk factors such as indoor pollution and the use of biomass fuel [7]. More than half of all rural residents lack access to critical healthcare [8] and many need to travel long distances to reach facilities. Patients may be reluctant to seek care due to travel costs, time expenditure and loss of potential income, resulting in healthcare-seeking delays [9] and limited access to specialist services and pulmonary rehabilitation [6, 10]. Lower health literacy [11] and socioeconomic status [12] may also influence the underutilisation of care and the persistence of unhealthy habits such as smoking [13]. The urban-rural divide ultimately results in a higher disease burden in remote communities, characterized by delayed diagnoses and higher hospitalization and mortality rates [12].

E-health refers to the use of electronic technologies in health care and has greatly expanded due to technological advances and the spread of mobile phones and other digital tools. Additionally, the COVID-19 pandemic introduced an urgent need to replace physical appointments, increasing the use of e-health solutions [14]. Importantly, e-health can be a cost-effective resource for diagnosis, monitoring, and health education [15], and telemonitoring has been shown to improve health utilization in rural settings [16].

In patients with chronic respiratory diseases, e-health interventions have been shown to reduce emergency department attendance [17–19], hospitalisation rates [17], the number of exacerbations [19] and to increase pharmacological treatment compliance and physical activity rates [19]. The use of e-health interventions may, therefore, help to reduce health disparities in remote populations living with these diseases [20–22]. Indeed, there is evidence that telehealth interventions are non-inferior for improving COPD self-management in rural areas [23]. However, beyond the effectiveness of e-health interventions, whether implementation is successful is likely context-specific, and a comprehensive understanding of factors influencing this process is paramount. The aim of this qualitative evidence synthesis (QES) was, therefore, to analyse the qualitative and mixed-methods literature on e-health interventions in people with chronic respiratory diseases in remote settings and thereby identify barriers and facilitators for implementation.

## Methods

### Study design and research question

The QES was structured according to Cochrane’s EPOC guidance and the ENTREQ reporting guidelines (checklist: Supplementary Table 1) [24, 25]. The PerSPE(c)TiF framework was used to define the research question (Supplementary Table 2) [26]. In brief, the aim of this QES was to explore any factors related to the successful implementation of e-health technologies in remote settings worldwide, for diagnosis and follow-up of chronic respiratory diseases from the perspective of patients, healthcare workers and other stakeholders.

### Search strategy

Scoping searches on PubMed were used to refine the search strategy. Three key domains (‘e-health’, ‘rural’ or ‘remote’ setting, and ‘chronic lung diseases’) were identified. With the help of a librarian, search terms and variations were generated and combined. The search was piloted in MEDLINE (Ovid).

Five databases were searched in April 2023: MEDLINE, CINAHL, Embase, Web of Science and PsycINFO. The full search strategy is shown in Supplementary Table 3. Additionally, relevant reviews were screened for additional articles. Grey literature was not searched.

### Study screening and selection

Citations were imported to Endnote version 20.3 on 21.4.2023, and duplicates were removed, followed by title/abstract screening and full-text review based on predefined inclusion and exclusion criteria (Table 1). Articles were screened by two authors (ES and EB) independently. In case of disagreement, the article was included in the full-text review. All qualitative and mixed-methods studies published in English describing diagnosis, monitoring, or treatment of patients with chronic respiratory diseases, defined according to the criteria of the American Lung Association [27], in remote and rural settings using e-health were included. We chose to include abstracts as well as full-text articles in order to obtain the most comprehensive data, as the chosen methodology of thematic synthesis is appropriate for relatively thin data, keeping the level of confidence in the data in mind when interpreting these. With regard to COVID-19, studies on diagnosis, monitoring or long-COVID were included, while studies on short-term treatment were not, as findings from the former were expected to be relevant for this QES.

**Table 1:** Inclusion and exclusion criteria

Criteria for selection	Inclusion criteria	Exclusion criteria
Type of article	Full-text articles or abstracts of primary research	Literature review, systematic review, QES
Methodology	Any qualitative or mixed-methods approach	Quantitative research without qualitative components
Study population	Patients of any age suffering from or suspected of chronic respiratory diseases including multi-morbidity patients, and/or their families; any stakeholder working on chronic respiratory disease e-health interventions including but not limited to healthcare workers, administrative personnel, policy makers, technology manufacturers, researchers.	Patients with conditions other than chronic respiratory conditions; stakeholders not working on chronic respiratory disease e-health interventions
Type of setting	Remote or rural settings (no specific geographic restrictions)	Non-remote settings
Type of Intervention	Any e-health intervention directed at the management of chronic respiratory disease patients (diagnostic process, follow up, monitoring and treatment)	Interventions without e-health components; e-health interventions not directed at chronic respiratory disease patients; multi-disease e-health interventions where findings for subgroup of chronic respiratory disease patients are not described
Timing	No time limit	
Language	English	Non-English language
Type of outcomes	Any qualitative outcomes related to factors influencing the use of e-health, including but not limited to: - User attitudes and satisfaction with the technologies - Factors for prolonged engagement - Factors hindering e-health implementation	Quantitative outcomes not accompanied by qualitative findings

### Quality assessment

The assessment of the methodological limitations was done according to the Critical Appraisal Skills Programme (CASP) tool for qualitative research to evaluate the methodological rigour of selected studies [28]. The CASP assessment is presented in Supplementary Table 4. The findings were also assessed for confidence according to GRADE CERQual method (Supplementary Table 5). CERQual takes into account four different factors: [1] methodological limitations, relating to the study design or conduct [2], coherence, assessing the fit between the primary study findings and review findings [3], adequacy, describing the overall richness and quantity of the data and [4] relevance, describing the extent to which the data is applicable and relevant to the review question [29]. After assessing each individual component, the overall confidence was judged by one author (ES) as high, moderate, or low.

### Data analysis and thematic synthesis

The RETREAT framework [30], taking into account the review question, epistemology, timeframe, resources, expertise, audience and types of data, guided the choice of a thematic synthesis approach (Supplementary Table 6). The selected articles were uploaded to Atlas.ti software (version 24.0.1, 2002–2024). Articles were coded sentence-by-sentence based on units of meaning, applied to both first and second-order constructs present in the results and discussion sections of papers, to capture both primary opinions and authors’ interpretations [31]. First-order constructs (quotes from participants in the original study) are presented in italics, and second-order constructs are presented in normal font. Descriptive themes were generated grouping similar codes into a hierarchical tree structure. Thematic synthesis was combined with an a priori ‘best fit’ framework approach [32, 33]. The themes were discussed between the authors (ES, FB, and EB) and existing frameworks were sought to guide the analysis and presentation of the data, ultimately identifying the Digital Health Equity Framework (DHEF) [34]. The DHEF draws on the leading health disparities framework. It includes key determinants of health at different levels and incorporates a digital environment domain which enabled us to classify and interpret factors related to implementation of e-health in remote locations. In order to identify themes, deductive coding was used applying the DHEF and an inductive coding process was utilised to capture novel insights. Aligning with the DHEF, themes were arranged according to their relevance to determinants of health at different levels: individual (e.g., patient or healthcare provider attitude towards technology), interpersonal (e.g., patient-tech-healthcare provider relationship), community (e.g., healthcare infrastructure), society (e.g., tech policies and data standards). An additional level was added relating to the intervention itself (e.g., technology characteristics, design quality and product presentation). Within the data, barriers and facilitators were identified, highlighting those unique to remote populations.

## Results

### Search results

**Fig. 1:**
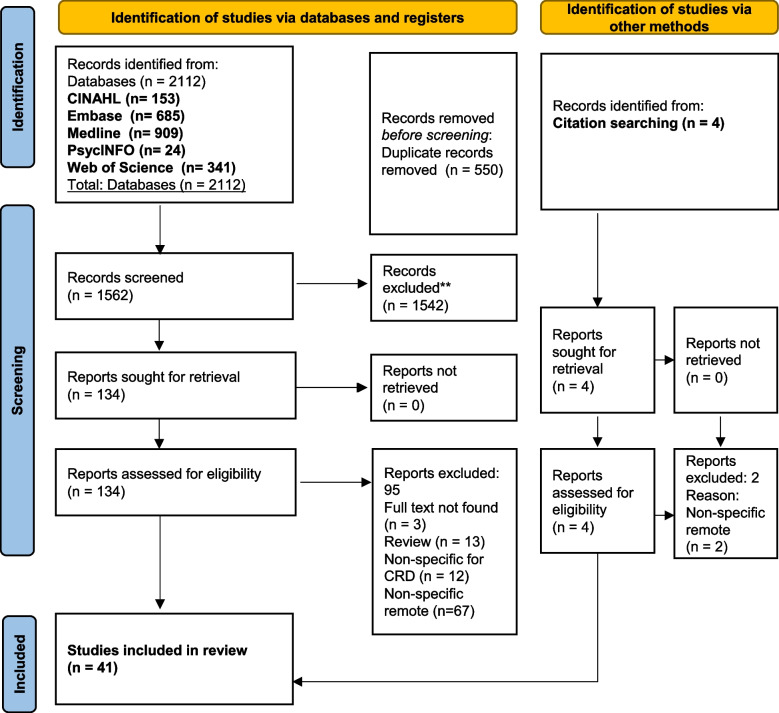
PRISMA Flowchart

After removing duplicates, 1562 articles were identified, with 134 studies sought for retrieval. Based on citation searching from relevant reviews, four additional studies were sought for retrieval. After full-text assessment, 41 studies were included (Fig. 1 and Table 2).

**Table 2:** – overview of selected articles

First author, year	Country, location	Setting	Type of publica-tion	Participants	Medical condition	E-health intervention	Study population			CERQual assess-ment of confidence in the evidence
							Age	% Female	Languages spoken	
Alexander, 2021 [35]	USA, North Carolina	McDowell, Mitchell, and Yancey counties, designated as Health Professional Shortage Areas for primary care and predominately rural	Qualitative research	Patients (*n* = 17)	COPD	Telehealth	Mean age 61 years +-SD 9.1	41	English	High
Alwashmi, 2019 [36]	Canada	Newfoundland and Labrador – area with substantial remote and rural population	Qualitative research	Nurses, physicians, pharmacists (*n* = 30)	COPD	Mobile health interventions	Mean age 39 years	NR	NR	High
Anticona, 2015 [37]	Peru	Rural areas in the Highlands and the Amazon basin	Qualitative research	Physicians (*n* = 30)	Multiple, including chronic respiratory diseases	Technological innovations, including telehealth and mobile health	Age 26–30 years	43	Spanish	Low
Boyd, 2007 [38]	Australia, Victoria	Five different rural Victorian towns	Mixed methods	Patients, developers, community members (8 focus groups of 4–10 participants)	Asthma	Multimedia education program	NR	NR	NR	Moderate
Brown, 2017 [39]	USA, North Dakota	Oakes, located in rural Dickey County	Mixed methods	Patients (*n* = 18)	Asthma	Telehealth for asthma education	NR	NR	NR	Moderate
Chaiyachati, 2013 [40]	South Africa, KwaZulu-Natal	Umzinyathi, a rural district of KwaZulu-Natal	Mixed methods	Mobile healthcare workers (*n* = 5)	Multidrug resistant TB	Mobile phone application	Median age 32 years (range 27–46)	20	English	Moderate
Chen, 2012 [41]	USA, West Virginia	Rural Appalachia	Abstract	Patients (*n* = 5)	Lung cancer	Nurse-interpreted telementoring intervention with patient ducation	Mean age 66 years +- 6.4	60	NR	Moderate
Concotelli, 2012 [42]	USA, New Mexico	NR	Abstract	Patients	CF	Telehealth adolescent support group	NR	NR	NR	Low
Cox, 2022 [43]	Australia, Victoria	33% of participants “from a rural location”	Abstract	Patients (*n* = 20)	Chronic respiratory diseases	Telerehabilitation	NR	NR	NR	Low
de Batlle, 2021 [44]	Spain	Lleida, a large rural area of over 4300 km^2^	Mixed methods	Patients (*n* = 76)	COPD	Mobile health-enabled integrated care model	Mean age 82 years	46	NR	Moderate
De San Miguel, 2013 [45]	Australia, Western Australia	Metropolitan area in Western Australia	Mixed methods	Patients (*n* = 80)	COPD	Home-based telehealth monitoring	Mean age 73 years, range 54–88)	52	English	Moderate
Demchenko, 2015 [46]	Canada, Saskatchewan	People living more than 100 km from Saskatoon	Abstract	Patients (*n* = 46)	Lung cancer	Nurse-clinical administered telehealth clinic	NR	NR	NR	Low
Douglas, 2013 [47]	Canada, Alberta	First Nations communities in Alberta	Mixed methods	Education instructors, phycisians, academics	Asthma	Self-management education curriculum	NR	NR	NR	Moderate
Ellington, 2021 [48]	Uganda, Jinja district	Two federally funded Ugandan primary care health facilities, one peri-urban and one rural	Qualitative research	Health care workers (*n* = 31)	Lower respiratory disease	Mobile health tool	*N* = 10 < 30 years; *n* = 11 30–40 years; *n* = 4 > 40 years	68	English and local languages	Moderate
Godden, 2011 [49]	UK, Scotland	Five participants “worked exclusively in remote and rural areas, while the others provided services across the region for urban, rural and remote patients”	Mixed methods	Phycisians, nurses, respiratory care professionals (*n* = 20)	COPD, Asthma, Lung cancer, OSAS	Telehealth	NR	65	NR	High
Goodridge, 2011 [50]	Canada, Alberta	“This health region encompasses an area of more than 40,000 km2, has population densities ranging from 1.1–2.0 persons per km2 and is designated as having no metropolitan influence by Statistics Canada.”	Qualitative research	Patients (*n* = 7)	COPD, Bronchiectasis	Not specified	Mean age 75 years (range 57–88)	71	English	Low
Guthrie, 2020 [51]	USA, Texas	“Northeast Texas, a 35-county area with a population close to 1.5 million, over half living in a rural area”	Abstract	Patients	CF	Telemedicine	NR	55	NR	Low
Hatem, 2022 [52]	USA, Massachusetts	NR	Abstract	Patients	COPD, COVID, interstitial lung disease	Telehealth-supported home based pulmonary rehabilitation program	Mean age 71 years	5	NR	Low
Johnson, 2021 [53]	USA, South Carolina	Four of nine schools were classified as rural	Mixed methods	Nurses, phycisians, respiratory therapist, program coordinator (*n* = 26)	Asthma	Mobile health application	25-34y *n* = 4; 35-44y *n* = 7; 45-54y *n* = 10; 55-64y *n* = 4; 65 + *n* = 1	96	NR	Moderate
Khan, 2017 [54]	Canada	Northern communities in Saskatchewan	Mixed methods	Phycisians	TB, various conditions	Multiple	NR	NR	NR	Low
Kok, 2023 [55]	Uganda, various districts	Rural communities, with a distance of at least 7 km to a health facility	Mixed methods	Community health workers (*n* = 24)	COVID	Telehealth	Mean age 38 years	90	Luganda, Lusoga, Runyankole	Moderate
Latycheva, 2013 [56]	Canada	Five First Nations and Inuit communities from across Canada	Mixed methods	Patients, parents, grandparents, community members, teachers	Asthma	Web-based asthma education materials	*N* = 14 6 to 12 years; *n* = 18 12 to 18 years; age of adults participants NR	NR	English	High
Locke, 2019 [57]	USA,	Rural patients, as defined by the US Census Bureau	Mixed methods	Patients	COPD, Asthma	Video telehealth inhaler training program	Mean age 69.2 years	0	NR	Moderate
Lundell, 2020 [58]	Sweden, Västerbotten county	A large and sparsely populated area with long distances to health care facilities	Qualitative research	Patients (*n* = 13)	COPD	Home telemonitoring system	NR	62	Swedish	Moderate
MacGeorge, 2021 [59]	USA, South Carolina	NR	Abstract	Nurses, teachers, administrators	Asthma	Telehealth-delivered school-based program	NR	NR	NR	Low
Mair, 1999 [60]	UK	NR	Mixed methods	Phycisians, Patients	COPD	Telecommunications technology	Mean age 62 years (range 52–72)	67	NR	Low
Mathur, 2019 [61]	India, Assam state	Five rural blocks from Darrang and Kamrup district of the Assam state	Qualitative research	Patients (*n* = 40)	TB	Outbound automated calls	Mean age 34 years, range 18–60	20	Assamese	Moderate
Mc Veigh, 2019 [62]	USA, Idaho	Large veteran hospital in Boise, Idaho, providing services to veterans located in mountainous regions of the county	Qualitative research	Nurses, phycisians, social workers (*n* = 16)	Non-malignant respiratory disease	Telemedicine/digital platform for palliative care	NR	NR	English	Moderate
McGee, 2020 [63]	USA, various states	Patients residing in rural/geographically isolated areas	Mixed methods	Patients (*n* = 49), informal caregivers (*n* = 49)	Chronic lower respiratory disease	Telehealth services	Patients: mean age 706 years	6.1	English	Moderate
Mendez, 2021 [64]	Chile, Santiago	Participants from any Chilean region	Mixed methods	Patients, physiotherapists (*n* = 22)	COPD	Mobile phone application	Median 37 (IQR 33–39 y)	50	Spanish	High
Musiimenta, 2020 [65]	Uganda, Southwestern	TB clinic within Mbarara Regional Referral Hospital in rural, southwestern Uganda	Mixed methods	Patients (*n* = 35), social supporters (*n* = 15)	TB	Mobile health intervention	Median 32 (patients); 37 (social supporters)	43 (patients); 60 (social supporters)	Runyankole	Moderate
Ng, 2014 [66]	Australia, South Australia	Port Augusta and Whyalla (300 and 381 km from Adelaide respectively) and Tennant Creek (500 km from Alice Springs)	Abstract	Patients	COPD, CF, OSAS	Telehealth consultations	NR	NR	NR	Low
Otty, 2023 [67]	Australia, Queensland	Regional tertiary public hospital in northern Australia, with a significant number of geographically dispersed rural and remote communities	Qualitative research	Patients, informal caregivers (*n* = 19)	Lung cancer	Telehealth consultations	Median 64 (IQR 52–82 y)	48	NR	Moderate
Petitte, 2014 [68]	USA, West Virginia	Patients’ homes in rural Appalachia	Mixed methods	Patients (*n* = 10)	Lung cancer	Home telemonitoring intervention	Median 66 (IQR 58–73)	50	NR	Low
Ratchakit, 2020 [69]	Thailand, Chiang Rai	TB clinic at Chiangrai Prachanukroh Hospital, a tertiary-care hospital located in Thailand’s northernmost province	Mixed methods	Patients (*n* = 80)	TB	Mobile health intervention	Mean 50 ± 16 (intervention arm)	40	Thai	Moderate
Raza, 2009 [70]	USA, Michigan/Wisconsin	Tertiary care hospital in Milwaukee and small rural hospitals in Iron Mountain (MI) and Appleton (WI) for veterans located in northern Wisconsin and the Upper Peninsula of Michigan	Mixed methods	Patients (*n* = 314)	Pulmonary specialist diagnostic consult	Telehealth consultations	NR	NR	NR	Moderate
Roberts, 2012 [71]	UK, Scotland	Sheltered housing in Oban and in a community hall on the Isle of Luing	Qualitative research	Patients, phycisians, nurses, nurse specialists, housing warden, project managers (n = NR)	COPD	Home and community telehealth monitors	NR	NR	NR	High
Ruseckaite, 2021 [72]	Australia, Victoria	NR	Abstract	Patients, informal caregivers (*n* = 15)	CF	Telehealth consultations	Mean 40.2 (patients); 41.8 (caregivers)	NR	NR	Low
Venter, 2012 [73]	New Zealand	Rural communities in Turangi and Taupo areas	Mixed methods	Patients (*n* = 20)	COPD	Home telemonitoring intervention	NR	NR	NR	Moderate
Wilson, 2022 [74]	USA, Midwest states	Rural areas across five Midwest states	Abstract	Phycisians, nurse practicioners, phycisian assistants (*n* = 10)	COPD	Telemedicine	NR	NR	NR	Low
Young, 2012 [75]	USA, Wisconsin	Family Health Center of Marshfield, serving 254 rural municipalities in north-central Wisconsin	Mixed methods	Patients (*n* = 98)	Asthma	Telepharmacy intervention	Mean 44.6 (SD 15.8)	75	English	High

*CF* cystic fibrosis, *COPD* chronic obstructive pulmonary disease, *NR* not reported, *OSAS* obstructive sleep apnea syndrome, *TB* tuberculosis

### Study characteristics and quality assessment

We included 11 primary qualitative studies, 21 mixed-methods studies, and 9 abstracts. The studies were published between 1999 and 2023. The geographical location varied; fifteen studies took place in the USA, six in Canada, six in Australia, three in Uganda, three in the UK, two in India and one in Peru, South Africa, Spain, Sweden, New Zealand, Chile and Thailand each (see Table 2 for details on the setting). Sixteen studies (39%) reported the language spoken by the participants, which was English in nine studies, Spanish in two studies, and Assamese, Swedish, Runyankole and Thai in one study each. One study reported that interviews and focus groups were done in English and ‘local languages’, and one study was done in Luganda, Lusoga and Runyankole. None of the studies reported the proportion of participants whose mother tongue was not English. Five studies explicitly mentioned the inclusion of Indigenous, First Nations or Aboriginal participants [47, 54, 56, 67, 73], two of which reported involvement of these groups in the study design itself, either in the form of an advisory group [47] or by partnering of the researchers with First Nations leaders [54]. Twelve studies included a mix of different chronic respiratory diseases, ten of the studies focused on COPD, seven on asthma, four on tuberculosis, four on lung cancer, three on cystic fibrosis and one on COVID-19 (Table 2).

The results of the CASP assessment of methodological limitations of the included studies are shown in Supplementary Table 4. According to the CERQual assessment of the findings, there were seven studies that inspired high confidence in their findings, while twenty instilled moderate confidence. The remaining fourteen studies elicited low confidence in their results (Table 2 and Supplementary Table 5). The reasons for low confidence ratings were diverse; notably, for several studies only abstracts were published without corresponding full articles, impacting the ability to assess the methodology and the richness and thickness of the findings.

### Description of themes

**Fig. 2:**
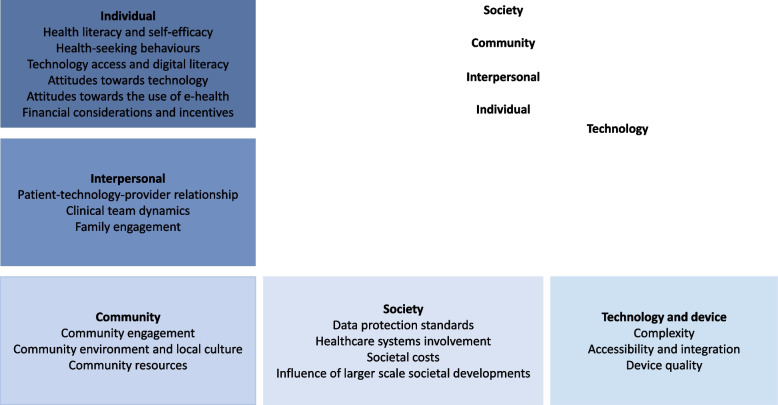
Graphical representation of the relevant themes at five different levels

Our synthesis, guided by the DHEF, yielded 19 themes across five levels, each with interrelated facilitators and barriers for the implementation of e-health interventions in remote locations. A graphical representation of the themes is shown in Fig. 2. The key facilitators and barriers identified through this QES are illustrated in Table 3.

**Table 3:** Overview of facilitators and barriers for e-health interventions associated with each level

Level	Facilitators	Barriers
Individual	• Patient willingness to become more autonomous, increase health literacy and self-management quality • Usability as educational tool • Patient familiarity with technology • Patient and HCP interest in learning new technologies • Scheduling flexibility and reduction of in-person appointments • No need for companion • Sense of reassurance with constant monitoring/access • Personalized care • Reduced travel costs, overall affordability • Cost-effectiveness	• May not be helpful unless integrated with behavioral change interventions • Patient age and poor digital literacy • Patient and HCP lack of knowledge about existing resources • Patient low literacy levels • Patient and HCP low interest in new technologies, technology perceived as challenging, skepticism • Preference for in-person consultation and fear of loss of human contact • Sense of hyperconnectivity • Increased workload and fear of not being adequately compensated (HCP) • Negative attitude towards tech devices (e.g., smartphones) • Steep set-up phase • Cost of device, lack of insurance
Interpersonal	• HCP can manage more patients • Reduced risk of infectious diseases transmission • Improved constant monitoring • Clinical team building and creation of multidisciplinary teams • Greater family involvement	• Reduced non-verbal communication • HCP feeling constantly available “on demand” • Sense of false security in the patient • Deterioration of relationships within pre-existing teams due to increased responsibilities
Community	• Community engagement • Use of role models • Cultural appropriateness • Availability of the translation in local languages • Focus on multiple conditions	• Poor networks • Lack of pre-existing health infrastructure • Focus on one disease at a time only
Society	• Lack of concern about personal health data privacy • Use of HIPAA compliant technologies • Trust in tech developer • Governmental and local leaders’ involvement • Private-public partnerships • Perceived as cost-saving • COVID-19 experience • General availability and diffusion of tech devices	• Risk of health data leakage • Lack of global privacy standards • Concerns around cost-effectiveness • Maintenance of the infrastructure • High training demand
Technology and device	• Ease of set-up and use • Easy interventions and advice • Translation in local language • Availability of tech support • Large fonts, audio support (e.g., for visually impaired) and recorded materials • Appealing graphic features, fun approach, customization • Portable, light devices	• Content and language complexity • Excessive length of interactive sessions • Unavailability in local language • Non-communicability between different software • Bulky, heavy devices • Difficult to clean

*HCP* healthcare provider, *HIPAA* Health Insurance Portability and Accountability Act

## Individual level factors

### Health literacy and self-efficacy

E-health requires a different form of patient participation than traditional consults. This participatory nature could positively influence self-efficacy and health literacy. Many healthcare providers (HCPs) [36, 71] and patients [47, 58, 69, 73, 75] felt that e-health facilitated autonomy and increased the quality of self-management. E-health was often used as a tool for education and was seen to have advantages compared to traditional in-person education, allowing for more detailed instruction [38, 39, 64, 75] over an extended period [36, 64, 75]. For patients, being able to review information at their convenience [36, 38, 64] was important. Additionally, using e-health, they gained a better comprehension of their own physiological reactions [39, 58, 75] and general health status [37, 58, 69, 71], leading to improved self-management of disease [39, 45, 58, 73].

“She was so proud of herself that she contacted the research team the next day to describe her successful self-management.” Oakes, North-Dakota [39].

Nonetheless, the view of e-health increasing self-efficacy was not universal. Whereas many patients found e-health helpful in interpreting their symptoms, some did not experience this benefit [58]. Some participants felt that e-health was taking responsibility away from them, as the HCP would monitor their parameters [58, 73]. Furthermore, some HCPs noted that in certain socio-economic contexts, a diagnostic/therapeutic e-health intervention was not sufficient as a standalone measure but should rather be integrated with behavioural change interventions [36].

### Health seeking behaviours

Despite the differing opinions on the ultimate impact on self-efficacy, one clear beneficial aspect of e-health interventions in remote locations was the positive impact these had on health-seeking behaviours among patients. HCPs recognized the value of e-health in reaching patients who might otherwise avoid traditional consults for various reasons, or who would have ignored early warning symptoms [39].

Similarly, e-health facilitated communication with patients who typically only seek medical attention during exacerbations or when prompted by family members:

*“some young asthmatic patients who don’t ever come and see me unless they’re exacerbated or that*,* you know*,* or their mothers phone me and say they’re not very well. for the youngsters that’s good. Even my asthmatic guy*,* who I hardly ever see*,* whenever I send him a text I always get a reply”.* Scotland [49].

### Technology access and digital literacy

While e-health had the potential to positively impact health-seeking behaviour, the benefits could be hampered by problems with training. Adequate training for both patients [35, 36, 43, 57, 58, 64] and HCPs [36, 37, 48, 71] emerged as a key factor to enable technology access and the implementation of e-health interventions [58]. Familiarity with technology, rather than age, was thought to be a key positive determinant of e-health adoption, suggesting that future generations of elderly individuals might be better equipped to utilize e-health solutions [48, 53]:

*“I had patients who are older than 90 who never owned a computer in their life and managed to do their sessions on their iPads and send it to me with no trouble. So*,* I think it depends on maybe education level and understanding*,* and maybe how things are explained to them.”* Canada, Newfoundland and Labrador [36].

Previous experiences with technology also influenced acceptance, with nurses familiar with smartphones showing greater comfort and likelihood of using new interventions compared to those less technologically savvy [61].

Nonetheless, the influence of advanced age on e-health adoption was widely recognized, with several studies noting that elderly patients tended to use e-health less frequently [36, 38, 53]. Some elderly patients reported struggling to learn how to use the devices [69], while HCPs expressed scepticism about their ability to do so [36, 53].

Another obstacle to the adoption of e-health within health systems was the lack of knowledge about existing e-health resources [36, 39, 56, 61, 71].

Additionally, lower levels of education were linked to a preference for audio over text materials due to low literacy levels, identified as a barrier to use in multiple studies [47, 56, 61].

### Attitudes towards technology

Related to the quality of training at the time of implementation, we noted that pre-existing attitudes towards technology also played a role. Both patients [58, 64] and HCPs [48] were more likely to use e-health when they had positive attitudes towards learning new technologies:

*“[Providers] usually like new technology*,* I think they will be excited to use it and therefore they are likely to download [the app]. In addition*,* people prefer digital information than opening and reading what is in the […] book.“* Uganda, Jinja district [48].

Conversely, negative attitudes towards technology acted as a barrier. In patients, the following were specific barriers for the acceptability of new technologies: lack of interest in technology [35], lack of motivation of the patient or the caregiver [36, 52, 53, 64], negative attitudes towards mobile phones [36, 48] and technology in general [36, 49, 58] or the “technologization” of society [58], not feeling comfortable with e-health or technology [35], perceiving it difficult to learn a technology [58], and a strong preference for in-person consults [39]. Some HCPs were also sceptical towards technology, expressing concerns that experienced physicians may be reluctant to change their way of practicing medicine [36].

### Attitudes towards the use of e-health interventions

The personal attitudes of patients and HCPs regarding e-health interventions varied greatly across settings and countries. Generally, patients were more likely to engage if they felt comfortable with e-health prior to use [35, 39, 75], saw it as convenient [35, 39, 43, 49, 51, 53, 61, 69], wanted to know more about their condition or health information or had a baseline interest in e-health [58].

Overall, e-health was perceived to be associated with several benefits. With some interventions, physical appointments were not needed, which made scheduling for patients easier and convenient [36].

“to go into a facility […] sometimes that’s really onerous for people, especially people who are suffering from COPD, so they’re going to have shortness of breath and exertion and find it even harder to get from the parking lot into the hospital” Canada, Newfoundland and Labrador [36].

Non-live interventions were especially acceptable, allowing patients to access care on a flexible schedule, accommodating those unable to take time off work during business hours [61]. This encouraged patients to seek help earlier (especially when experiencing debilitating symptoms [35, 36, 64]), reduced travel costs [64], increased independence due to lack of need for a companion [64] and allowed provision of care during times when a trip was not possible, e.g. due to weather conditions [35].

Some HCPs felt encouraged that the interventions gave patients more responsibility and made them more accountable, as their actions were tracked. This led to higher therapy adherence and better outcomes [36, 64, 69]. Some patients felt reassured by being monitored 24 h a day and receiving personalized care [61, 64].

Nonetheless, numerous sources also reported negative attitudes towards the use of e-health technologies. Patients’ and HCPs’ negative feelings arose primarily from a sense of hyperconnectivity, increased workload [49, 58, 71], and scepticism about the reliability of devices and efficacy of the interventions [36, 58, 60, 63]. Many patients and HCPs reported a sense of threat over the risk of losing human contact and face-to-face clinical interactions [35–37, 49, 67]. In one study, this was described as feeling “chained” to the digital environment:

“[…] since thoughts about the system and remembering to perform the tests were always present. This could lead to a feeling of being “fed up” and a wish for “a break” from the system” Sweden, Västerbotten county [58].

Some HCPs thought that their reliance on their mobile phones would affect the way patients view them, believing it is rather a distraction or a tool to compensate for lack of knowledge, and could act as a barrier to use [48]. Some HCPs feared that they would lose their current autonomy by being tracked and followed through standardised interventions [71]. Devices could also display false abnormalities, such as low oxygen saturation from cold fingers, causing unnecessary anxiety and doubt— issues typically avoided when an HCP would interpret results in traditional consultations [58].

Many HCPs complained that their workload for the same number of patients increased due to extra tasks and information to review and retain [36, 44, 71], especially in the set-up phases of a new intervention [48].

Individual patient characteristics also contributed to shaping attitudes in a negative way. For example, the severity of health conditions could affect the feasibility: pulmonary rehabilitation [52] and e-health interventions requiring physical exercise were viewed by some patients as unacceptably demanding [58, 63].

Evidence of the efficacy of e-health for improving care was seen as important for both patients as well as for HCPs:

*“ […] I think that type of evidence is what’s going to change my mind as a practitioner about whether it’s worth using it or not.”* Canada, Newfoundland and Labrador [36].

“more information justifying the use of the telemonitoring system was needed, since the purpose of using it was unclear” Sweden, Västerbotten county [58].

### Financial considerations and incentives

The use of e-health was facilitated for patients when the equipment was free [64] or at least affordable [36], for example by saving on commutes [64]. For HCPs, the use of e-health was facilitated if the intervention was perceived as cost-effective for the healthcare facilities they were working in [36, 44]. Conversely, high costs of electronic devices [48, 53, 64] or use of personal devices and mobile data [37, 40, 53], the need to share devices, such as a mobile phone, due to the high cost of acquisition of own device [61], and lack of insurance [36, 51] represented barriers to the use of e-health interventions:

“A nurse with experience conducting mHealth programs endorsed this concern by adding that only about 10% of participants may remain in the mHealth intervention if the insurance company stopped paying for the service.” Canada, Newfoundland and Labrador [36].

Uncertainty about costs caused scepticism and reluctance to engage in e-health. When the participants had little information about actual costs, they feared that new interventions would be expensive [35].

A significant disincentive was the lack of financial rewards, where the HCP would feel not adequately compensated for using e-health [36, 44], even though it brought clinical benefits:

“a physician insisted: ‘*I mean we’re all so busy that nobody wants to do anything for free because why would I do that for free if I get paid for it.’ ”* Canada, Newfoundland and Labrador [36].

## Interpersonal level factors

The following level of our framework captures the internal dynamics of the healthcare teams and health facilities, and relationships with and between patients and their families, influencing the feasibility and acceptability of the interventions.

### Patient-technology-provider relationship

E-health significantly impacted patient-HCP relationships and communication patterns, with reduced face-to-face interaction emerging as a key sub-theme. This allowed HCPs to manage more patients [36, 71], decreased the need for physical infrastructure [36], and, during the COVID-19 pandemic, reduced the risk of transmission [64]. However, this also resulted in negative feelings among both patients and HCPs, as remote visits were felt to affect nonverbal communication [49] and decrease interpersonal connection [35, 36, 48, 49].

*“I like to have a bit of actual contact and eye contact*,* and hear the tone of someone’s voice*,* and a gentle touch sometimes can be so reassuring*,* you know. I think it’s going to be lost with this type of technology.”* Canada, Newfoundland and Labrador [36].

Nonetheless, some noted that e-health allowed for the maintenance of good communication if the patient and HCP already knew each other [35]. Indeed, many participants expressed the need [36, 37, 64, 72] or preference [35, 36, 64] to continue face-to-face contact in some form, even when e-health was used [72].

The idea of being connected 24/7 created expectations in some cases, and some HCPs expressed concerns about the constant need to be always on demand:

*“[a patient] would often e-mail about his condition […] with questions that required careful consideration and reference to the notes*,* so it was actually pretty labour intensive without really much or any associated clinical benefit”* Scotland [49].

Frequent contact also resulted in a feeling that patients were being better monitored by HCPs [58, 73], but on the other hand, was criticized by HCPs for potentially giving a false sense of security [58].

### Clinical team dynamics

Within organisations, e-health could facilitate contact between HCPs. For example, pharmacists could connect with physicians [35] and physicians could connect with specialists not normally easily reachable such as pulmonologists, pulmonary rehabilitation experts and palliative care physicians [37, 49, 62]. This seemed to be the case in remote areas specifically, as often only a primary care physician was available to offer care, and the patient was unable or unwilling to move to a hospital with specialised care:

*“I dream of a system where the primary care docs (doctors in the community) get to phone a friend and the palliative care physician does the consultation with the primary care provider”* Rural America [62].

Team members were also able to communicate with each other, building cohesion and optimising care for patients:

*“With [this phone] we are able to work as a team*, […] *it has made communication between the team quite good and also for the patients because if you don’t communicate as a team.they are getting less care than they should.’’* KwaZulu-Natal, Umzinyathi district [40].

However, in one study, the increased workload and shifting of care needs associated with the e-health intervention resulted in the deterioration of staff cohesion as new skills and extra responsibilities became necessary [71].

### Family engagement

E-health did not only affect communication inside HCP-teams and between HCPs and patients. The possibility to view information at home through a device allowed for greater family involvement in education [53, 56] and therapy [61, 73].

Engaging with the family was important, especially with paediatric patients, as parent involvement was required for participation [59].

#### Community level factors

The third level is represented by community factors. Aspects of the local settings and community health facilities played an important role, as did local culture.

### Community resources

The availability of a reliable internet or phone connection represented an important enabler to the use of e-health. Local network issues resulting in poor audio connection [61], or ‘freezing’ of the app and upload issues [40] led to underuse and delays in patient care.

Some studies noted that the greatest trigger for introducing e-health was the absence of pre-established infrastructure or services. Examples included places without access to basic health resources [61], and locations where specialist services were unavailable [36, 62].

### Community engagement

Proper design and planning of the e-health intervention, including community engagement, was seen as essential. Engaging patients [38, 44] and HCPs [44] during the development and planning process improved the usability of technologies.

“The feedback [from human-computer interface testing] resulted in language changes, directional aids, greater use of graphics and age-specific content.” Australia, Victoria [38].

Community engagement was equally important in the implementation phases. One study noted that once the programme was functioning, additional patients should be recruited by a local familiar HCP or another trustworthy stakeholder [56].

“study participants […] conveyed that uptake of tele-COPD programs may be greater if tele-health visits were described and demonstrated to patients by their own health care provider or other trusted individual(s).” USA, North Carolina [35].

### Community environment and local culture

Adaptation to local cultures and conditions was important for intervention acceptability. One study commented on the cultural significance of tobacco use within the community, highlighting the challenge of addressing health-related habits when these are embedded in local culture [47]. Another example involves the perception of symptoms, which may differ across cultures and populations [41]. Because this review included papers from various locations, specific aspects varied. However, some common themes emerged. Two studies discussed the importance of including aspects from local culture, including local (non-western) remedies [37, 56] and spiritual aspects [56]:

“one participant of this study described his own uncommon approach to treat patients in his […] community by integrating traditional and Western medical practices. He felt this approach helped him to enhance his relationship with the community and increase the overall compliance with medical procedures.” Peru, Highlands and Amazon basin [37].

To make the interventions more appropriate for local communities, two studies discussed the need to include some examples that are relevant or meaningful for the local cultural context, highlighting the importance of cultural appropriateness especially in indigenous communities:

“examples of more relevant content included stories of Aboriginal people living with asthma, and information on housing issues (eg mould), as well as using different languages and visual learning formats (eg appropriate coloring, characters, images, and pictures).” First Nations and Inuit communities in Canada [56].

“Specific adaptations to [the intervention] included incorporating […] the imagery of the circle; the seven sacred teachings; and inclusion of elders, family members and the community. […] The wolf was recommended as a culturally relevant mascot based on its symbolism as a teacher and member of a larger community, and for its capacity to howl, which was symbolic of strong lungs.” Canada, Alberta [47].

“[patients] recommended adding environmental factors common to Aboriginal communities (eg road dust, forest fire smoke) as relevant information to newly developed materials” First Nations and Inuit communities in Canada [56].

One crucial part of adapting interventions was translation into local languages [44, 56]:

*“A lot of kids are adopted by a grandparent and their education is very low to none and they often don’t speak English . educational materials need to be in different languages for people who do not speak or read English and French.”* Community health worker; First Nations and Inuit communities in Canada [56].

Beyond cultural considerations, opinions varied on the best adaptation approach for different patient populations and conditions. Some favoured single-condition interventions, while others, especially caregivers of multimorbid patients, preferred interventions addressing multiple conditions [48].

*‘‘I think the phones would be helpful if not only looks at TB. It should also look at other diseases or problems.like immunizations and ante-natal care.’’* KwaZulu-Natal, Umzinyathi district [40].

## Societal level factors

The fourth level of the framework is represented by societal factors influencing the implementability of e-Health interventions.

### Data protection standards

Universally for various communities, health information privacy played a large role. Depending on the setting, health data privacy [36, 40] and transparency of data [58] were not seen as barriers for the utilization of e-health interventions:

“Sending data regarding health parameters electronically was considered quite harmless: *‘There’s information that is more sensitive.But I mean this*,* so what? I’ve got COPD*,* okay*,* lots of people know that.’ ”* Sweden, Västerbotten county [58].

Some noted that e-health even offered increased privacy compared to paper-based practice [40].

*‘‘we lose [papers] and we lost them under the car seats or on the floor and you find them everywhere.[the phone] is always in your pocket.’’* KwaZulu-Natal, Umzinyathi district [40].

One study cited the credibility of the technology developer to play a significant role in the privacy concerns [36]. On the other hand, several studies reported on participants’ fears about private health information unwanted disclosure [35, 36, 58, 63, 71], as electronic communication was seen at risk of cyberattacks and data leakage [58]. It should also be noted that privacy standards are not universal and may be associated with cultural values, representing potential barriers to the use of e-health interventions:

“A cultural belief referred to as “Appalachian pride,” could be a barrier for tele-health adoption. Appalachian pride is comprised of multiple cultural values (e.g., privacy) that are upheld in tight-knit communities. Appalachian pride may impact tele-health adoption and preferences.” USA, North Carolina [35].

### Healthcare systems involvement

Governmental health system involvement was seen as necessary to improve trust and coordination. Coordination through government [35, 48, 49] could facilitate the implementation and scaling-up of the intervention.

*“. the one thing that will make it (tele-health) have more chance of success is if NHS Highland do set up infrastructure to support the delivery of that”* Scotland [49].

It was also acknowledged that the government and local policy leaders had the power to block the wider use of the intervention, and their engagement was crucial:

“The administrator explained that the Ministry of Health in Uganda and local district health officials would need to approve the app prior to large scale distribution across public and private facilities. Additionally, support at these leadership levels will be critical for widespread uptake and implementation of [the intervention].” Uganda, Jinja district [48].

Regardless of cost-effectiveness, it was thought that the scaling up of any intervention involving multiple stakeholders needed to be coordinated at the governmental level [44]. Other providers were sceptical that an e-health program would be implementable without a total overhaul of the local healthcare systems [37].

### Societal costs

If implemented correctly, e-health was seen as potentially saving costs at the societal level through decreased use of other healthcare resources [36]. Nonetheless, some users were concerned about the cost of implementation [49] and maintenance of infrastructure [36]:

“there is the initial cost of establishing the infrastructure, including costs related to storing data in the cloud. In addition, costs related to the maintenance and replacement of outdated technology were discussed[.]*”* Canada, Newfoundland and Labrador [36].

It was suggested that the partnership of private entities with public ones could bring down costs [36, 44]:

“some of this could be outsourced to private entities that are already doing this type of work, thereby reducing expenses to taxpayers.” Canada, Newfoundland and Labrador [36].

### Influence of larger scale societal developments

The COVID-19 pandemic proved to be an effective facilitator for the use of e-health. Some studies cited that because of the pandemic, the number of clinic visits was reduced and replaced by e-health [35, 51, 64]. This was attributed to the desire to decrease contagion [51, 64], as well as the belief that e-health was more accessible than in-person consults [72].

General technological development was also noted as an external factor affecting e-health. As technology advanced and became more widespread in society, its use in medicine in the form of e-health also became more acceptable, reducing barriers such as confusion about compensation [39], complexity [48] and privacy [39]:

“With current advances in secure, HIPAA-compliant, web- based systems with billing capabilities, it is now possible for more health professionals to consider expansion of their practice to include this type of care for patients with asthma and other disease states.” USA, North Dakota [39].

Several points relating to the future of e-health were discussed. It was expected that as the use of e-health becomes more widespread, the costs would come down due to economies of scale. While some doubted that local health systems would be ready to implement e-health [62], and demands for training and infrastructure may be too high [37], others thought scaling up e-health may also improve patient outcomes as care becomes more standardised [71].

“Large-scale implementation may realize benefits in standardization of practice and economies of scale” Scotland [71].

## Technology and device level factors

Finally, the fifth level corresponds to intrinsic technology and device characteristics reported to increase or decrease the implementability of the interventions.

### Complexity

It was seen as important that the devices and technologies were not too complicated [48], easy to set up , to learn [58], and to use [36, 58, 64, 71], as well as reliable [40, 58, 70].

Many studies highlighted a preference for simple and clear language [36, 38, 56, 64].

“One nurse […] thought the language should be *“set at a grade six reading level*,* so there’s no issues with comprehension of what they’re being asked or told.”* Canada, Newfoundland and Labrador [36].

Some patients felt that the advice was particularly useful when it pertained to achievable and easy interventions:

“Majority of the participants preferred information that was immediate and easy to implement, i.e., nutrition, effects of tobacco and alcohol, and government schemes.*”* India, Darrang and Kamrup districts [61].

Preference for simple, relevant, easy-to-follow advice, such as for example interactive maps for home visits, was not only limited to patients but also echoed by some HCPs [40, 44].

On the other hand, overly complex systems were seen as a barrier to continued use, both for patients [36, 58] as well as for HCPs [36, 37]. Several studies noted that also contents complexity, as well as excessive session duration, led to the underuse of the interventions:

*“A participant said*,* “If the call comes while I am busy working*,* I will not listen to it if it is long. So I want the call to be shorter than 5 min.”* India, Darrang and Kamrup districts [61].

“most participants identified materials that were ‘too busy’ with ‘too much writing’.” First Nations and Inuit communities in Canada [56].

“Interpreting the test results was also perceived as difficult, including both the numbers and the charts, which made it difficult to notice if there had been any changes” Sweden, Västerbotten county [58].

Related to complexity was the ability to solve problems when they emerge. Two studies recognised that there would inevitably be technical problems with e-health. The provision of technical support was recommended [36, 40].

### Accessibility and integration into existing systems

Integration of the e-health technology into existing systems was found to be important. Some found that e-health was underused because different setups prevented different technologies from communicating, and it was, therefore, challenging to integrate new products into daily practice [36, 44]:

“not achieving a full integration with legacy EMR systems and the coexistence of 2 management systems (usual care and IC) at the same time (which implied some duplicity of tasks) were the main barriers to adoption.” Spain, Lleida [44].

The importance of accessibility features, such as large fonts [36, 44, 48, 61, 69] loud voices [69], audio components for the visually impaired and text for the hearing impaired [38], was emphasized as their absence hindered utilization for patients with corresponding disabilities. For e-health equipment utilizing visual displays like computer or mobile applications, a visually appealing graphic user interface was favoured across several studies [36, 38, 39, 48, 56, 64]. Graphics were found to engage users and bolster long-term adherence [56]. Reminders and notifications were explicitly preferred and linked to improved medication adherence [44, 64, 65, 69]. Some studies highlighted the preference for recording and reflecting on the given advice [58, 61].

*“I would have liked the option to record the call. I would have recorded the call and listened to it again whenever I wanted to.”* India, Darrang and Kamrup districts [61].

### Device quality

Device portability and cleanliness were key factors affecting feasibility and acceptability. Bulky, heavy devices discouraged mobility [58, 60, 69, 73], and difficulty in cleaning after shared use was cited as a deterrent [36].

Device quality mattered significantly; poor-quality devices, particularly older ones, were deemed useless [60]. While many studies indicated a preference for clinic-supplied devices [48, 64, 66], one study reported that using personal smartphones over separate devices increased the likelihood of utilizing interventions such as mobile apps [48].

There was also little consensus on how the information should be presented, as the studies were concerned with different populations and conditions, not comparing approaches. One study covering educational resources for paediatric populations indicated that a fun approach was preferred:

“Our results are consistent, revealing the need for children to ‘be taught in a fun way by doing activities’ and the need for personal interaction with parents.” First Nations and Inuit communities in Canada [56].

Customization based on user preferences [40, 48] and available resources [36, 44, 48, 71] emerged as the key consideration.

## Discussion

This QES identified various factors relating to the effective implementation of e-health interventions for chronic respiratory diseases in remote locations. In general, e-health interventions were well received but also carried a number of concerns and limitations that could reduce their effectiveness. Moreover, e-health was deemed insufficient as a standalone intervention and should be integrated into existing care practices [35–37, 58, 64, 72].

E-health was identified by many patients living in remote communities and HCPs as a tool with the potential to overcome the obstacle of distance to health facilities, reducing commutes and related costs, and expanding access to care. Increased self-efficacy and health literacy were additional benefits at the individual level. Concerns and barriers were noted across all levels. At the community level, concerns related to lack of telecommunications and healthcare infrastructure— hence, while e-health was perceived as a solution to certain resource limitations, other resource limitations undermined its potential. The main concerns at the interpersonal level related to the reduction of in-person interactions and fear of losing personal connections. Additionally, HCPs identified additional burdens for the staff, negative attitudes towards technology, and poorly planned implementation processes as barriers to successful adoption.

Our findings are in line with previously published reviews [76, 77], but some specifically relevant issues are worth highlighting. Adapting e-health interventions to the local setting was seen as especially relevant in remote and rural environments [35, 40, 47, 55, 56, 62, 65, 68]. Some studies suggested that integrating aspects important to the community, such as symbolism [47, 56], traditional medical practices [37] and habits specific to the location [56] with ‘Western’ medical information was appreciated, leading to higher adoption. By contrast to cultural factors, considerations about the availability of e-health or applications in the preferred language of the patient/community were discussed in very few papers, highlighting an important gap in the evidence base.

In addition to the setting, the target population is also key to consider at an early stage since the feasibility of e-health interventions may vary between patient groups. For example, patients in poor health with advanced disease might be physically unable to self-measure or undergo pulmonary rehabilitation without in-person assistance [52, 58, 63]. Another aspect of this was described in a meta-ethnography on telemedicine in COPD: while patients with high disease burden experienced an increased need for telemedicine, patients in a stable period or at an early phase of the illness perceived the disease as becoming too prominent with telemedicine, and a need to be more detached [76]. Familiarity with technology in general and training needs may also differ between groups, as we identified longer learning times for patients with lower education levels and advanced age [39, 61, 69].

Financial concerns were identified as significant barriers across various levels in the majority of studies. A clear understanding of financial aspects is crucial for the successful implementation of e-health initiatives. For instance, patients should be assured that e-health services are covered through insurance or government support. The importance of financial aspects was mirrored by a systematic review of patient satisfaction with telehealth demonstrating the need to provide additional means of reimbursement from the government in order to make telehealth available [78].

It is worth noting that the term ‘e-health’ includes a wide variety of interventions, and although the choice for a specific e-health modality was not the focus of this QES, many papers discussed their reasoning behind this. Individual preferences varied: some users preferred mobile-phone-based interventions and apps [35], others preferred video interaction over phone calls [51, 67], some wanted live interactions with immediate feedback [56, 64, 69, 73, 75], others preferred non-live communication done at the convenience of the patient [61], some liked group-based interventions [42, 43], others wanted graphical aspects rather than text or audio [56] and yet others expressed the desire to combine different modalities [39, 48, 58]. While some studies described the preference for combining modalities, such as video consults with devices for measuring physical parameters, others cautioned against it, warning that multiple electronic systems from different manufacturers may not communicate [36, 44]. However, while individual preferences emerged, none of the studies focused on comparing different types of e-health interventions, and assessing the advantages and disadvantages of different modalities is beyond the scope of this paper.

This study has a number of limitations. First, there is a risk of over- or underestimating e-health acceptability and feasibility barriers due to selection bias in the primary research, preferentially including individuals with explicit views or motivations towards e-health. Second, it is challenging to generalize from studies undertaken from a wide range of contexts—across countries, populations, languages, continents etc—given the impact of setting- and population-specific factors; nevertheless, we found themes common across settings. Third, many studies focused on the initial phases of the implementation, potentially magnifying perceptions related to technology novelty while overlooking factors influencing longer-term use and adherence. Moreover, geographical representation poses a limitation, with an overrepresentation of Global North viewpoints. Certain populations (e.g. non-English speakers) were also underrepresented in the included papers, which may further limit generalizability of our findings. Additionally, while we aimed to include various patient populations, there was a higher number of studies on COPD and asthma compared to cystic fibrosis and other chronic diseases, potentially limiting the generalizability of findings. Lastly, there’s the possibility of publication bias, wherein less impactful qualitative research might not have been published [79], potentially leading to an incomplete representation of viewpoints. We recognize that e-health is a rapidly evolving field, and our findings will need ongoing validation against emerging evidence, technological advances, and changes in digital policy as they become available.

Despite these limitations, this synthesis may have some implications for both practice and research. Our findings suggest that a functional infrastructure that can respond to changing demands is a prerequisite for the effective use of e-health. When considering a new intervention, all stakeholders should be involved in the design and implementation process. Informing patients about the content and goal, benefits and limitations of the intervention is essential to avoid miscommunication and to support willingness to engage [58]. Even though customization may be more labour- and cost-intensive when compared to the use of generic interventions, attention should be paid to local and cultural aspects relating to language, beliefs and attitudes, as failure to do so may result in a lack of use in the longer term. General digital literacy was found to be an important factor relating to the use of specific e-health interventions and providing education to increase this could increase the adoption and use of e-health. Some level of governmental involvement is required to ensure compatibility of systems, adequate resources and equitable distribution of devices. Interesting areas for future research, beyond the scope of this synthesis, include comparing various e-health modalities and focusing on a specific disease under the umbrella of chronic respiratory disease, as well as comparing our findings with the use of e-health in non-respiratory diseases. Further research should include underrepresented populations and consider new technological and societal developments.

## Conclusions

This synthesis identified several factors that can affect the successful implementation of e-health in remote and rural locations for chronic respiratory disease patients. These factors can be used to inform the design and implementation of future e-health interventions. Intervention objectives, target population, geographical location, telecommunication and health care infrastructure capacity, local culture, language, and norms, and available human resources should be carefully considered to optimise the interventions for the best outcomes. Relevant stakeholders must be consulted throughout the process to produce an appropriate and feasible intervention.

## Supplementary Information

Supplementary Material 1.

## Data Availability

The dataset used during the current study is available from the corresponding author upon reasonable request.

## Abbreviations

CASP: Critical Appraisal Skills Programme
COPD: chronic obstructive pulmonary disease
DHEF: Digital Health Equity Framework
EMR: electronic medical record
HCP: healthcare provider
TB: tuberculosis
QES: qualitative evidence synthesis
