# Supplementary material for: Facilitators and barriers affecting the implementation of e-health for chronic respiratory diseases in remote settings: a qualitative evidence synthesis
Source: BMC Health Serv Res. 2025 Jan 4;25:19. doi: 10.1186/s12913-024-12050-4 (PMC11699737; doi:10.1186/s12913-024-12050-4)
Supplement: Supplementary file 1 — Supplementary Material 1. [file 12913_2024_12050_MOESM1_ESM.docx]

**Supplementary material**

**Supplementary Table 1- ENTREQ checklist**

| **Item** | **Guide and description** | **Reported on page #** |
| --- | --- | --- |
| Aim | To analyse the qualitative and mixed-methods literature on e-health interventions in people with chronic respiratory diseases in remote settings and thereby identify barriers and facilitators for implementation. | 6 |
| Synthesis methodology | Thematic synthesis was chosen. The aim of this QES is to contribute to existing theory and explore the topic using previously used models, applied in a novel way to a previously unexplored combination of topic, setting and patient-population. | 6-8 |
| Approach to searching | A pre-planned search strategy was implemented, aiming to identify all relevant studies. | 5& supplementary table 3 |
| Inclusion criteria | Primary qualitative or mixed-methods research. Studies looking at remote and rural locations were included, without geographical limits. No limits were set on year of publication, country of origin or study type. | 5, Table 1 |
| Data sources | Pubmed, CINAHL, Embase, Web of Science and PsycINFO were searched for articles. These databases were chosen to gain perspectives on implementation and attitudes related to the studied topic. | 5 |
| Electronic Search strategy | See supplementary information – table 3 | Supplementary table 3 |
| Study screening methods | The resulting articles were screened by two authors independently (ES and EB) based on title and abstract. Additionally, ES screened relevant reviews for additional articles. In cases of disagreements, the article was included in the full-text review. | 7 |
| Study characteristics | See table 2 | Table 2 |
| Study selection results | See figure 1. | 9 & Figure 1. |
| Rationale for appraisal | Using the CERQual tool we assessed methodological limitations, coherence, adequacy and relevance. | 9-10 |
| Appraisal items | The assessment of the methodological limitations was done according to the Critical Appraisal Skills Programme (CASP) tool for qualitative research to evaluate the methodological rigour of selected studies. The findings were also assessed for confidence according to GRADE CERQual method. | 9-10 |
| Appraisal process | The appraisal was done by one author (ES). | 7-8 |
| Appraisal results | See supplementary tables 5 and 6. | Supplementary tables 5 and 6 |
| Data extraction | Articles were coded sentence-by-sentence based on units of meaning, applied to both first and second-order constructs present in the results and discussion sections of papers, to capture both primary opinions and authors’ interpretations. | 8 |
| Software | Atlas.Ti was used for coding. | 8 |
| Number of reviewers | Coding was done by two authors (ES and FB) | 8 |
| Coding | Coding was done according to units of meaning, where one author doing the initial coding and the other regrouping of existing codes. | 8 |
| Derivation of themes | Themes were derived by a combination of inductive and deductive methods. Descriptive subthemes were generated grouping similar codes into a hierarchical tree structure. Thematic synthesis was combined with an a priori ‘best fit’ framework approach. The themes were discussed between the authors (ES, FB, and EB) and existing frameworks were sought to guide the analysis and presentation of the data, ultimately identifying the Digital Health Equity Framework (DHEF). | 8 |
| Study comparison | Subsequent studies were coded into pre-existing concepts, and new concepts were created when deemed necessary. | 8 |
| Quotations | This has been done throughout the manuscript. | 10-35 |
| Synthesis output | This has been done in the discussion of the manuscript. | 35-39 |

**Supplementary Table 2- PerSPE(c)TiF research question formulation**

| **Per** | **S** | **P** | **E** | **(C)** | **Ti** | **F** |
| --- | --- | --- | --- | --- | --- | --- |
| **Perspective** | **Setting** | **Phenomenon of interest/problem** | **Environment** | **Comparison** | **Timing** | **Findings** |
| From the perspective of patients, health care workers and other stakeholders | In the setting of remote locations worldwide | Successful implementation of e-health technologies | Within environments with distantly located health-care facilities | - | Starting from the diagnostic process, and including the subsequent follow-up | Any factors related to the successful implementation of these technologies. |

## Supplementary Table 3 – Search strategy – (Ovid Medline syntax)

| **Number** | **Theme** | **Term type** | **Medline Ovid search term** | **EMBASE Ovid Search term** | **PsycINFO Ovid search term** | **CINAHL search term** | **Web of Science search term** |
| --- | --- | --- | --- | --- | --- | --- | --- |
| 1 | Remote setting | Free search term | ("Tribal*" OR "tribe*" OR "indigenous*" OR "aboriginal*" OR "rural*" OR "population densit*" OR nomad* OR frontier* OR ((remote* OR distant*) adj3 locat*) OR ((remote* OR distant* OR isolat*) adj3 (village* OR settlement* OR camp* OR communit*))).ti,ab. | ("Tribal*" OR "tribe*" OR "indigenous*" OR "aboriginal*" OR "rural*" OR "population densit*" OR nomad* OR frontier* OR ((remote* OR distant*) adj3 locat*) OR ((remote* OR distant* OR isolat*) adj3 (village* OR settlement* OR camp* OR communit*))).ti,ab. | ("Tribal*" OR "tribe*" OR "indigenous*" OR "aboriginal*" OR "rural*" OR "population densit*" OR nomad* OR frontier* OR ((remote* OR distant*) adj3 locat*) OR ((remote* OR distant* OR isolat*) adj3 (village* OR settlement* OR camp* OR communit*))).ti,ab. | ("Tribal*" OR "tribe*" OR "indigenous*" OR "aboriginal*" OR "rural*" OR "population densit*" OR nomad* OR frontier* OR ((remote* OR distant*) N3 locat*) OR ((remote* OR distant* OR isolat*) N3 (village* OR settlement* OR camp* OR communit*))) | ("Tribal*" OR "tribe*" OR "indigenous*" OR "aboriginal*" OR "rural*" OR "population densit*" OR nomad* OR frontier* OR ((remote* OR distant*) NEAR/3 locat*) OR ((remote* OR distant* OR isolat*) NEAR/3 (village* OR settlement* OR camp* OR communit*))) |
| 2 | Remote setting | Mesh term | Rural Population/ OR Rural Health/ OR Rural Health Services/ OR Hospitals, Rural/ OR Regional Medical Programs/ | rural health/ OR rural population/ OR rural area/ OR rural hospital/ OR rural health care/ | exp Rural Health/ OR exp Rural Environments/ | (MH "Rural Health Personnel") OR (MH "Rural Health Centers") OR (MH "Hospitals, Rural") OR (MH "Rural Health Services") OR (MH "Frontier Nursing Service") OR (MH "Rural Health") OR (MH "Services for Australian Rural AND Remote Allied Health") OR (MH "Association for Australian Rural Nurses") OR (MH "Rural Areas") OR (MH "Rural Health Nursing") OR (MH "Australian Rural Nurses AND Midwives") OR (MH "Rural Population") | - |
| 3 | Chronic respiratory disease | Mesh term | exp Lung Diseases/ | exp lung disease/ | exp Lung Disorders/ | (MH "Lung Diseases+") | - |
| 4 | Chronic respiratory disease | Free search term | (((lung* or "pulmonary" or "respiratory") adj3 (disease* or condition*)) or "COPD" or "chronic obstructive pulmonary disease" or "chronic obstructive airways disease*" or "asthma" or "cystic fibrosis" or "pulmonary hypertension" or "pulmonary rehabilitation" or spirom* or inhal* or "interstitial lung disease" or "ILD" or "Emphysema" or "Bronchitis" or "Bronchiectasis" or "Lung cancer" or pulmonolog* or dyspn* or tubercul*).ti,ab. | (((lung* or "pulmonary" or "respiratory") adj3 (disease* or condition*)) or "COPD" or "chronic obstructive pulmonary disease" or "chronic obstructive airways disease*" or "asthma" or "cystic fibrosis" or "pulmonary hypertension" or "pulmonary rehabilitation" or spirom* or inhal* or "interstitial lung disease" or "ILD" or "Emphysema" or "Bronchitis" or "Bronchiectasis" or "Lung cancer" or pulmonolog* or dyspn* or tubercul*).ti,ab. | (((lung* or "pulmonary" or "respiratory") adj3 (disease* or condition*)) or "COPD" or "chronic obstructive pulmonary disease" or "chronic obstructive airways disease*" or "asthma" or "cystic fibrosis" or "pulmonary hypertension" or "pulmonary rehabilitation" or spirom* or inhal* or "interstitial lung disease" or "ILD" or "Emphysema" or "Bronchitis" or "Bronchiectasis" or "Lung cancer" or pulmonolog* or dyspn* or tubercul*).ti,ab. | TI ( ((lung* OR pulmonary OR respiratory) N3 (disease* OR condition*) OR COPD OR "chronic obstructive pulmonary disease" OR "chronic obstructive airways disease" OR "asthma" OR "cystic fibrosis" OR "pulmonary hypertension" OR "pulmonary rehabilitation" OR spirom* OR inhal* OR "interstitial lung disease" OR "ILD" OR "Emphysema" OR "Bronchitis" OR "Bronchiectasis" OR "Lung cancer" OR pulmonolog* OR dyspn* OR tubercul* ) OR AB ( ((lung* OR pulmonary OR respiratory) N3 disease*) OR COPD OR "chronic obstructive pulmonary disease" OR "chronic obstructive airways disease" OR "asthma" OR "cystic fibrosis" OR "pulmonary hypertension" OR "pulmonary rehabilitation" OR spirom* OR inhal* OR "interstitial lung disease" OR "ILD" OR "Emphysema" OR "Bronchitis" OR "Bronchiectasis" OR "Lung cancer" OR pulmonolog* OR dyspn* OR tubercul* ) | ((lung* OR pulmonary OR respiratory) NEAR/3 disease*) OR COPD OR "chronic obstructive pulmonary disease" OR "chronic obstructive airways disease" OR "asthma" OR "cystic fibrosis" OR "pulmonary hypertension" OR "pulmonary rehabilitation" OR spirom* OR inhal* OR "interstitial lung disease" OR "ILD" OR "Emphysema" OR "Bronchitis" OR "Bronchiectasis" OR "Lung cancer" OR pulmonolog* OR dyspn* OR tubercul* |
| 5 | eHealth | Free search term | (("Internet-based" adj3 ("care" or program*)) or "telemedicine" or "tele-medicine" or "telehealth" or "tele-health" or "e-health" or "ehealth" or "mobile health" or "mobile healthcare" or "mhealth" or "m-health" or "digital health" or "virtual medicine" or "Internet" or "web" or "telecommunication" or "SMS" or "tele-monitor" or "telemonitor" or "telemanagement" or "tele-management" or "teleconsultation" or "tele-consultation" or "telecare" or "tele-care" or "telematic" or "telenurse" or "tele-nurse" or "remote consult" or "wireless" or "bluetooth" or "tele-homecare" or "telehomecare" or "remote care" or "tele-support" or "telesupport" or "computer mediated therap*" or "virtual medicine").ti,ab. | (("Internet-based" adj3 ("care" or program*)) or "telemedicine" or "tele-medicine" or "telehealth" or "tele-health" or "e-health" or "ehealth" or "mobile health" or "mobile healthcare" or "mhealth" or "m-health" or "digital health" or "virtual medicine" or "Internet" or "web" or "telecommunication" or "SMS" or "tele-monitor" or "telemonitor" or "telemanagement" or "tele-management" or "teleconsultation" or "tele-consultation" or "telecare" or "tele-care" or "telematic" or "telenurse" or "tele-nurse" or "remote consult" or "wireless" or "bluetooth" or "tele-homecare" or "telehomecare" or "remote care" or "tele-support" or "telesupport" or "computer mediated therap*" or "virtual medicine").ti,ab. | (("Internet-based" adj3 ("care" or program*)) or "telemedicine" or "tele-medicine" or "telehealth" or "tele-health" or "e-health" or "ehealth" or "mobile health" or "mobile healthcare" or "mhealth" or "m-health" or "digital health" or "virtual medicine" or "Internet" or "web" or "telecommunication" or "SMS" or "tele-monitor" or "telemonitor" or "telemanagement" or "tele-management" or "teleconsultation" or "tele-consultation" or "telecare" or "tele-care" or "telematic" or "telenurse" or "tele-nurse" or "remote consult" or "wireless" or "bluetooth" or "tele-homecare" or "telehomecare" or "remote care" or "tele-support" or "telesupport" or "computer mediated therap*" or "virtual medicine").ti,ab. | (Internet-based N3 (care OR program*)) OR "telemedicine" OR "tele-medicine" OR "telehealth" OR "tele-health" OR "e-health" OR "ehealth" OR "mobile health" OR "mobile healthcare" OR "mhealth" OR "m-health" OR "digital health" OR "virtual medicine" OR "Internet" OR "web" OR "telecommunication" OR "SMS" OR "tele-monitor" OR "telemonitor" OR "telemanagement" OR "tele-management" OR "teleconsultation" OR "tele-consultation" OR "telecare" OR "tele-care" OR "telematic" OR "telenurse" OR "tele-nurse" OR "remote consult" OR "wireless" OR "bluetooth" OR "tele-homecare" OR "telehomecare" OR "remote care" OR "tele-support" OR "telesupport" OR "computer mediated therapy" OR "virtual medicine" | ("Internet-based" NEAR/3 ("care" OR program*)) OR "telemedicine" OR "tele-medicine" OR "telehealth" OR "tele-health" OR "e-health" OR "ehealth" OR "mobile health" OR "mobile healthcare" OR "mhealth" OR "m-health" OR "digital health" OR "virtual medicine" OR "Internet" OR "web" OR "telecommunication" OR "SMS" OR "tele-monitor" OR "telemonitors" OR "telemanagement" OR "tele-management" OR "teleconsultation" OR "tele-consultation" OR "telecare" OR "tele-care" OR "telematic" OR "telenurses" OR "tele-nurse" OR "remote consult" OR "wireless" OR "bluetooth" OR "tele-homecare" OR "telehomecare" OR "remote care" OR "tele-support" OR "telesupported" OR "computer mediated therapy" OR "virtual medicine" |
| 6 | eHealth | Mesh term | exp Telemedicine/ | exp telemedicine/ OR exp telehealth/ | exp Telemedicine/ | (MH "Telemedicine+") OR (MH "Telehealth+") OR (MH "Telerehabilitation") | - |
| 7 | Remote setting |  | 1 OR 2 | 1 OR 2 | 1 OR 2 | 1 OR 2 | 1 OR 2 |
| 8 | Chronic respiratory disease |  | 3 OR 4 | 3 OR 4 | 3 OR 4 | 3 OR 4 | 3 OR 4 |
| 9 | eHealth |  | 5 OR 6 | 5 OR 6 | 5 OR 6 | 5 OR 6 | 5 OR 6 |
| 10 | Combining results |  | 7 AND 8 AND 9 | 7 AND 8 AND 9 | 7 AND 8 AND 9 | 7 AND 8 AND 9 | 7 AND 8 AND 9 |

## Supplementary table 4- Methodological limitations of studies based on the Critical Appraisal Skills Program (CASP) methodology

| **First author** | **Was there a clear statement of the aims of the research?** | **Is a qualitative methodology appropriate?** | **Was the research design appropriate to address the aims of the research?** | **Was the recruitment strategy appropriate to the aims of the research?** | **Was the data collected in a way that addressed the research issue?** | **Has the relationship between researcher and participants been adequately considered?** | **Have ethical issues been taken into consideration?** | **Was the data analysis sufficiently rigorous?** | **Is there a clear statement of findings?** | **How valuable is the research?** | **Overall assessment of methodological limitations** |
| --- | --- | --- | --- | --- | --- | --- | --- | --- | --- | --- | --- |
| *Alexander* | *Yes* | *Yes* | *Yes* | *Yes* | *Yes* | *No* | *Yes* | *Yes* | *Yes* | *highly* | *Minor* |
| *Alwashimi* | *Yes* | *Yes* | *Yes* | *Yes* | *Yes* | *Yes* | *Yes* | *Yes* | *Yes* | *highly* | *Minor* |
| *Anticona* | *Yes* | *Yes* | *No* | *No* | *Yes* | *Unclear* | *Yes* | *Yes* | *Yes* | *moderate* | *Moderate* |
| *Boyd* | *Yes* | *No* | *No* | *No* | *Yes* | *Unclear* | *Yes* | *Yes* | *No* | *moderate* | *Moderate* |
| *Brown* | *No* | *No* | *No* | *Unclear* | *No* | *Unclear* | *Yes* | *No* | *Yes* | *moderate* | *Moderate-Severe* |
| *Chaiyachati* | *Yes* | *Yes* | *Yes* | *Yes* | *Yes* | *No* | *Yes* | *Yes* | *Yes* | *quite* | *Minor* |
| *Chen* | *No* | *No* | *No* | *Yes* | *Yes* | *Unclear* | *Unclear* | *No* | *Yes* | *low* | *Moderate-Severe* |
| *Concotelli* | *Yes* | *No* | *No* | *Yes* | *Yes* | *Unclear* | *Unclear* | *Unclear* | *No* | *low* | *Moderate-Severe* |
| *Cox* | *Yes* | *Yes* | *Yes* | *Unclear* | *Yes* | *Unclear* | *Unclear* | *Unclear* | *Yes* | *moderate* | *Moderate-severe* |
| *de Batlle* | *Yes* | *No* | *Yes* | *Yes* | *Unclear* | *Yes* | *Yes* | *Yes* | *Yes* | *moderate* | *Minor-Moderate* |
| *De San Miguel* | *Yes* | *No* | *No* | *Yes* | *Yes* | *Yes* | *Yes* | *No* | *Yes* | *moderate* | *Moderate* |
| *Demchenko* | *No* | *No* | *No* | *Yes* | *Unclear* | *Unclear* | *Unclear* | *Unclear* | *No* | *low* | *Severe* |
| *Douglas* | *Yes* | *No* | *Yes* | *Yes* | *Yes* | *Yes* | *Yes* | *Yes* | *Yes* | *highly* | *Minor-Moderate* |
| *Ellington* | *Yes* | *Yes* | *Yes* | *No* | *Yes* | *Unclear* | *Yes* | *Yes* | *Yes* | *highly* | *Minor-moderate* |
| *Godden* | *Yes* | *Yes* | *Yes* | *Yes* | *Yes* | *Unclear* | *Yes* | *Yes* | *Yes* | *moderate* | *Minor* |
| *Goodridge* | *No* | *Yes* | *No* | *No* | *Yes* | *Unclear* | *Yes* | *Yes* | *Yes* | *moderate* | *Moderate-Severe* |
| *Guthrie* | *No* | *No* | *No* | *Unclear* | *Unclear* | *Unclear* | *Unclear* | *Unclear* | *Yes* | *low* | *Severe* |
| *Hatem* | *No* | *No* | *No* | *Unclear* | *No* | *Unclear* | *Unclear* | *Unclear* | *Yes* | *low* | *Severe* |
| *Johnson* | *Yes* | *Yes* | *Yes* | *Unclear* | *Yes* | *No* | *No* | *Yes* | *Yes* | *moderate* | *Minor-Moderate* |
| *Khan* | *No* | *No* | *No* | *Unclear* | *Unclear* | *Unclear* | *No* | *No* | *No* | *low* | *Severe* |
| *Kok* | *Yes* | *Yes* | *Yes* | *Yes* | *Yes* | *Unclear* | *Yes* | *Yes* | *Yes* | *moderate* | *Minor* |
| *Latycheva* | *Yes* | *Yes* | *Yes* | *Yes* | *Yes* | *Unclear* | *No* | *Yes* | *Yes* | *highly* | *Minor* |
| *Locke* | *Yes* | *No* | *No* | *No* | *Yes* | *Yes* | *Unclear* | *Yes* | *Yes* | *moderate* | *Moderate* |
| *Lundell* | *Yes* | *Yes* | *Yes* | *Yes* | *Yes* | *Unclear* | *Yes* | *Yes* | *Yes* | *highly* | *Minor* |
| *MacGeorge* | *Yes* | *Yes* | *Yes* | *Unclear* | *Unclear* | *Unclear* | *Unclear* | *Unclear* | *Yes* | *moderate* | *Severe* |
| *Mair* | *No* | *No* | *Yes* | *Unclear* | *Yes* | *Unclear* | *No* | *No* | *Yes* | *low* | *Moderate-Severe* |
| *Marthur* | *No* | *Yes* | *Yes* | *Yes* | *Yes* | *No* | *No* | *No* | *Yes* | *moderate* | *Moderate* |
| *McVeigh* | *Yes* | *Yes* | *Yes* | *Unclear* | *Yes* | *Unclear* | *Yes* | *Yes* | *Yes* | *moderate* | *Minor-Moderate* |
| *McGee* | *Yes* | *No* | *No* | *Yes* | *Yes* | *Unclear* | *Unclear* | *No* | *Yes* | *moderate* | *Moderate-Severe* |
| *Mendez* | *Yes* | *Yes* | *Yes* | *Yes* | *Yes* | *Unclear* | *Yes* | *Yes* | *Yes* | *highly* | *Minor* |
| *Musiimenta* | *Yes* | *Yes* | *Yes* | *Unclear* | *Yes* | *Unclear* | *Yes* | *Yes* | *Yes* | *moderate* | *Minor-moderate* |
| *Ng* | *No* | *No* | *No* | *Unclear* | *Yes* | *Unclear* | *Unclear* | *Unclear* | *Yes* | *low* | *Severe* |
| *Otty* | *Yes* | *Yes* | *Yes* | *No* | *Yes* | *Yes* | *Yes* | *Yes* | *Yes* | *moderate* | *Minor* |
| *Petitte* | *Yes* | *No* | *No* | *Yes* | *Yes* | *Unclear* | *No* | *No* | *Yes* | *moderate* | *Moderate-Severe* |
| *Ratchakit* | *Yes* | *Yes* | *Yes* | *No* | *Yes* | *Unclear* | *Yes* | *Yes* | *Yes* | *moderate* | *Minor-Moderate* |
| *Raza* | *Yes* | *No* | *Yes* | *Yes* | *Yes* | *No* | *Yes* | *Yes* | *Yes* | *moderate* | *Minor-moderate* |
| *Roberts* | *Yes* | *Yes* | *Yes* | *No* | *Yes* | *Unclear* | *Yes* | *Yes* | *Yes* | *highly* | *Minor* |
| *Ruseckaite* | *No* | *Yes* | *Yes* | *Unclear* | *Yes* | *Unclear* | *Unclear* | *Unclear* | *Yes* | *moderate* | *Severe* |
| *Venter* | *Yes* | *No* | *No* | *Yes* | *Yes* | *Unclear* | *Yes* | *No* | *Yes* | *moderate* | *Moderate* |
| *Wilson* | *Yes* | *Yes* | *Yes* | *Yes* | *Unclear* | *Unclear* | *Unclear* | *Unclear* | *Yes* | *low* | *Moderate-Severe* |
| *Young* | *Yes* | *Yes* | *Yes* | *Yes* | *Yes* | *No* | *Yes* | *Yes* | *Yes* | *moderate* | *Minor* |

## Supplementary table 5 - CERQual assessment of confidence in the evidence

| **First author** | **Coherence of data** | **Explanation low coherence** | **Adequacy of data** | **Explanation low adequacy** | **Relevance of data** | **Explanation relevance** | **CERQual assessment of confidence in the evidence** | **Explanation of CERQual assesment** |
| --- | --- | --- | --- | --- | --- | --- | --- | --- |
| Alexander | No or minor concerns | - | No or minor concerns | - | High | Highly relevant | High confidence | - |
| Alwashimi | No or minor concerns | - | No or minor concerns | - | High | Highly relevant | High confidence | - |
| Anticona | Moderate concerns | Consideration of other interventions in combination to eHealth | Moderate concerns | - | No-Minor | Inclusion of multiple conditions, not specific to eHealth | Low Confidence | Due to severe concerns about relevance and moderate concerns about methodological limitations, coherence and adequacy |
| Boyd | No or minor concerns | - | Moderate concerns | - | Minor-moderate | From viewpoint of developers, sources of information not clear | Moderate confidence | Due to moderate concerns about methodological limitations adequacy and relevance |
| Brown | No or minor concerns | - | Moderate concerns | - | Minor-moderate | Surveys, mostly quantitative | Moderate confidence | Due to moderate concerns about methodological limitations adequacy and relevance |
| Chaiyachati | No or minor concerns | - | No or minor concerns | - | Moderate | Includes non-respiratory TB patients | Moderate confidence | Due to moderate concerns about relevance |
| Chen | No or minor concerns | - | Severe concerns | Abstract only, mostly quantitative | Minor-moderate | Thin poor data | Moderate confidence | Due to severe concerns about adequacy and moderate concerns about methodological limitations and relevance |
| Concotelli | No or minor concerns | - | Severe concerns | Abstract only, mostly quantitative | No-Minor | Thin poor data | Low Confidence | Due to severe concerns about relevance and adequacy and moderate concerns about methodological limitations |
| Cox | No or minor concerns | - | Severe concerns | Abstract only | High-Moderate | Thin data | Low Confidence | Due to severe concerns about adequacy and moderate concerns about methodological limitations |
| de Batlle | No or minor concerns | - | Moderate concerns | - | Moderate | Includes also post-arthroplasty patients | Moderate confidence | Due to moderate concerns about methodological limitations, relevance and adequacy |
| De San Miguel | No or minor concerns | - | Moderate concerns | - | Moderate | Backgroundof patients (rural/remote) unclear | Moderate confidence | Due to moderate concerns about methodological limitations, relevance and adequacy |
| Demchenko | Moderate concerns | Technology used differs from other studies (limited interactivity) | Severe concerns | - | No-Minor | Abstract only, mostly quantitative | Low Confidence | Due to severe concerns about relevance, methodological quality and adequacy and moderate concerns about coherence |
| Douglas | No or minor concerns | - | Moderate concerns | - | Moderate | Includes literature review, not clear what information is primary | Moderate confidence | Due to moderate concerns about methodological limitations, relevance and adequacy |
| Ellington | No or minor concerns | - | No or minor concerns | - | No-Minor | Includes acute respiratory diseases as well | Moderate confidence | Due to severe concerns about relevance and moderate concerns about methodological limitations |
| Godden | No or minor concerns | - | No or minor concerns | - | High | Includes various health conditions | High confidence | - |
| Goodridge | Moderate concerns | Seemingly limited use of eHealth by patients | Moderate concerns | - | No-Minor | Not exclusive to eHealth | Low Confidence | Due to severe concerns about relevance and moderate concerns about methodological limitations, relevance and adequacy |
| Guthrie | No or minor concerns | - | Severe concerns | Abstract only | Minor-moderate | Poor data | Low Confidence | Due to severe concerns about methodological limitations and adequacy and moderate concerns about relevance |
| Hatem | No or minor concerns | - | Severe concerns | Abstract only, mostly quantitative | No-Minor | Thin poor data | Low Confidence | Due to severe concerns about methodological limitations and relevance and moderate concerns about adequacy |
| Johnson | No or minor concerns | - | No or minor concerns | - | High | Highly relevant | Moderate confidence | Due to moderate concerns about methodological limitations |
| Khan | Moderate concerns | Covers many modalities of eHealth superficially | Severe concerns | Thin data, provenance of data not clear | Minor-moderate | Relevant topic, poor thin data | Low Confidence | Due to severe concerns about methodological limitations and adequacy and moderate concerns about relevance and coherence |
| Kok | Moderate concerns | Focus on COVID; includes also non-chronic patients | No or minor concerns | - | No-Minor | Focus more on COVID than eHealth | Moderate confidence | Due to severe concerns about relevance and moderate concerns about coherence |
| Latycheva | No or minor concerns | - | No or minor concerns | - | High | Highly relevant | High confidence | - |
| Locke | No or minor concerns | - | Moderate concerns | - | Minor-moderate | Measures satisfaction, mostly quantitative | Moderate confidence | Due to moderate concerns about methodological limitations relevance and adequacy |
| Lundell | No or minor concerns | - | No or minor concerns | - | High | Highly relevant | Moderate confidence | - |
| MacGeorge | No or minor concerns | - | Severe concerns | Abstract only | Minor-moderate | Relevant topic, poor thin data | Low Confidence | Due to severe concerns about relevance and adequacy and moderate concerns about methodological limitations |
| Mair | Serious concerns | Outdated technology, problems have changed | Moderate concerns | - | No-Minor | Old study, outdated technology | Low Confidence | Due to severe concerns about methodological limitations and coherence and moderate concerns about relevance and adequacy |
| Marthur | No or minor concerns | - | Moderate concerns | - | High-Moderate | Includes non-respiratory TB patients | Moderate confidence | Due to moderate concerns about relevance and adequacy |
| McVeigh | No or minor concerns | - | Moderate concerns | - | Moderate | Not exclusive to e-health | Moderate confidence | Due to moderate concerns about methodological limitations relevance and adequacy |
| McGee | Moderate concerns | Focus on effects of COVID-pandemic | Moderate concerns | - | Moderate | Survey data, mostly quantitative | Moderate confidence | Due to moderate concerns about methodological limitations, relevance, coherence and adequacy |
| Mendez | No or minor concerns | - | No or minor concerns | - | High-Moderate | Unclear how many patients were from a rural setting | High confidence | - |
| Musiimenta | Moderate concerns | Use of SMS-reminders, non-interactivity | Moderate concerns | - | No-Minor | Not specific to e-health | Moderate confidence | Due to severe concerns about relevance and moderate concerns about methodological limitations, coherence and adequacy |
| Ng | No or minor concerns | - | Severe concerns | Abstract only, thin data | Minor-moderate | Mostly quantitative data, relatively thin | Low Confidence | Due to severe concerns about methodological limitations and adequacy and moderate concerns about relevance |
| Otty | Moderate concerns | Focus on treatment pathway; e-health only one part | Moderate concerns | - | No-Minor | Not specific to e-health | Moderate confidence | Due to severe concerns about relevance and moderate concerns about coherence and adequacy |
| Petitte | Moderate concerns | Use of telemonitoring; non-interactivity | Severe concerns | Poor, thin quality evidence | No-Minor | Limited qualitative data on e-health included | Low Confidence | Due to severe concerns about methodological limitations and adequacy and moderate concerns about relevance |
| Ratchakit | No or minor concerns | - | Moderate concerns | - | Moderate | Includes non-respiratory TB patients | Moderate confidence | Due to moderate concerns about methodological limitations adequacy and relevance |
| Raza | No or minor concerns | - | Severe concerns | Mostly quantitative, thin data | Moderate | Limited qualitative data included | Moderate confidence | Due to severe concerns about adequacy and moderate concerns about methodological limitations and relevance |
| Roberts | No or minor concerns | - | No or minor concerns | - | High | Highly relevant | High confidence | - |
| Ruseckaite | No or minor concerns | - | Severe concerns | Abstract only, thin data | High-Moderate | Abstract only, relevant data | Low Confidence | Due to severe concerns about methodological limitations and adequacy |
| Venter | No or minor concerns | - | Moderate concerns | - | Moderate | Inclusion of congestive heart failure, mostly quantitative data | Moderate confidence | Due to moderate concerns about methodological limitations relevance and adequacy |
| Wilson | No or minor concerns | - | Severe concerns | Abstract only, thin data | No-Minor | Thin poor data | Low confidence | Due to severe concerns about relevance and adequacy and moderate concerns about methodological limitations |
| Young | No or minor concerns | - | No or minor concerns | - | High-Moderate | Mostly quantitative data, relatively thin | High confidence | - |

## Supplementary table 6 – Use of the RETREAT framework

| **Domain** | **Elaboration** |
| --- | --- |
| Review question | Broad, descriptive question aiming to identify any factors related to the successful implementation of e-health approaches. |
| Epistemology | Critical realism |
| Time/timeframe | Starting from the diagnostic process, and including the subsequent follow-up. |
| Resources | Externally funded project, with a team of a graduate student and post-doctoral researchers. |
| Expertise | General qualitative research skills. Access to a university librarian. |
| Audience and purpose | Potentially used in combination with primary research to implement future interventions. |
| Type(s) of data | Any qualitative and mixed-methods research, regardless of the methodology. The expected number of studies was expected to be moderate, conceptually relatively poor but contextually rich. |
| Choice of method | Thematic synthesis with ’Best Fit Framework’ approach |
| Justification of choice | The aim of this QES is to contribute to existing theory and explore the topic using previously used logic models, that are applied in a novel way to a previously unexplored combination of topic, setting and patient-population. Thematic synthesis is chosen. |
